# The Role of HMB Supplementation in Enhancing the Effects of Resistance Training in Older Adults: A Systematic Review and Meta-Analysis on Muscle Quality, Body Composition, and Physical Function

**DOI:** 10.3390/nu17223624

**Published:** 2025-11-20

**Authors:** Alberto García-Alonso, Juan Luis Sánchez-González, Víctor Navarro-López, Roberto Méndez-Sánchez, Luis Polo-Ferrero

**Affiliations:** 1 Department of Nursing and Physiotherapy, University of Salamanca, 37007 Salamanca, Spain; 2 Institute of Biomedical Research of Salamanca (IBSAL), 37007 Salamanca, Spain; 3 Department of Medicine, University of Salamanca, 37007 Salamanca, Spain; 4 Department of Physical Therapy, Occupational Therapy, Rehabilitation and Physical Medicine, Faculty of Health Sciences, Universidad Rey Juan Carlos, 28922 Madrid, Spain

**Keywords:** aging, β-hydroxy β-methylbutyrate, resistance training, body composition

## Abstract

Background/Objectives: Resistance training (RT) is a key strategy to counteract age-related declines in muscle strength and physical function. β-hydroxy-β-methylbutyrate (HMB) has been proposed as a complementary supplement to enhance these adaptations. However, the additional effects of RT plus HMB (RT+HMB) compared with RT alone remain unclear. This systematic review and meta-analysis evaluated the effects of RT+HMB versus RT alone on body composition, muscle quality (MQ), and physical function in older adults. Methods: Following PRISMA guidelines (PROSPERO: CRD420251144810), six databases (PubMed, Scopus, Cochrane Library, CINAHL, Web of Science, and ScienceDirect) were searched up to July 2025. Randomized controlled trials comparing RT+HMB with RT alone were included. Methodological quality was assessed with the PEDro scale and risk of bias using Cochrane RoB 2. Pooled standardized mean differences (SMDs) with 95% confidence intervals (CIs) were calculated. Results: Ten trials (*n* = 596) met inclusion criteria. RT+HMB produced modest and borderline significant improvements in handgrip strength (SMD 0.24; 95% CI 0.00–0.48; *p* = 0.05) and moderate benefits in Short Physical Performance Battery (SPPB) scores (SMD 0.54; 95% CI 0.12–0.95; *p* = 0.01). No significant effects were observed for gait speed, appendicular lean mass, MQ, fat mass, or body weight (*p* > 0.05). Five trials (50%) were rated at high risk of bias, limiting confidence in pooled estimates. Conclusions: HMB supplementation combined with RT may yield modest improvements in functional performance, particularly handgrip strength and overall physical function, without statically significant effects in body composition and MQ. Further high-quality RCTs are warranted to confirm its clinical relevance.

## 1. Introduction

Aging is a complex, multidimensional process characterized by progressive biological alterations that lead to declines in skeletal muscle mass, strength, and functional capacity [1,2]. Within this context, the concept of successful aging emphasizes the maintenance of physical and cognitive function, autonomy, and quality of life alongside longevity; however, its global prevalence remains low and varies considerably across populations [3].

The progressive loss of muscle mass and function is a central determinant of frailty, disability, and mortality in older adults [4,5,6]. Moreover, excess adiposity and elevated body mass index (BMI) have been consistently associated with poorer functional performance, including lower SPPB scores and reduced gait speed [2]. With advancing age, muscle quality (MQ) declines due to impaired protein synthesis, slower contraction velocity, altered neuromuscular activation, and increased fibrosis and intramuscular fat infiltration [7,8,9]. Although a universally accepted definition is lacking, the European Working Group on Sarcopenia in Older People 2 (EWGSOP2) described MQ as encompassing both micro- and macroscopic alterations in muscle architecture and composition, as well as muscle function per unit of mass—integrating histological, imaging, metabolic, and functional dimensions [10,11]. The Global Leadership Initiative on Sarcopenia (GLIS) has emphasized the need to standardize this concept, as using the term without specifying its components may generate ambiguity. In this context, muscle-specific strength, defined as muscle strength normalized to muscle size, has been proposed as a more precise descriptor of MQ [11,12].

Physical exercise and increased physical activity, together with nutritional interventions, appear to be the most effective strategies to counteract the physiological effects of aging, including frailty and sarcopenia, and to enhance functional capacity in older adults [13,14]. No validated pharmacological strategies currently exist to effectively prevent functional decline in older adults [15,16]. Currently, no validated pharmacological interventions exist to effectively prevent functional decline or to treat sarcopenia, and available evidence remains insufficient to support the clinical use of pharmacotherapy for obesity in this population [16,17].

Among exercise modalities, high-intensity resistance training (RT) has proven to be one of the most effective and safe strategies to improve muscle strength, muscle quality (MQ), and functional capacity in older adults, effectively counteracting age-related declines in muscle mass and performance [1,18,19,20]. Regular and progressive activation of skeletal muscles through structured RT enhances neuromuscular efficiency, contractile function, and independence in activities of daily living [21,22]. These effects are particularly evident with high-velocity RT, which has been associated with favourable changes in body composition and physical performance [19,23].

From a nutritional perspective, leucine supplementation has demonstrated strong evidence for promoting muscle mass gains in individuals with sarcopenia [13]. β-hydroxy-β-methylbutyrate (HMB), a leucine-derived metabolite, exerts multiple biological actions, including enhanced recovery following high-intensity exercise [13,24]. Available in calcium (HMB-Ca) and free acid (HMB-FA) forms, this compound is thought to stimulate protein synthesis via modulation of the growth hormone (GH) and insulin-like growth factor 1 (IGF-1) axis, promoting increases in muscle mass, lean mass, and strength—effects particularly relevant in older adults [24,25].

Nevertheless, substantial methodological heterogeneity exists across studies, with diverse exercise protocols [26], nutritional interventions [27], and participant characteristics [25,28] contributing to inconsistent findings. While some studies have compared RT alone with RT combined with nutritional supplementation, definitive conclusions remain elusive due to the distinct physiological mechanisms of each nutrient [27]. Others have evaluated the isolated or combined effects of exercise and protein-based supplementation, including HMB, on MQ and muscle function [5,28]. However, variations in exercise protocols or the absence of exercise in control groups introduce potential confounders, given the profound influence of physical activity on nutritional outcomes [29,30]. Previous meta-analyses have typically examined either HMB supplementation or RT in isolation, focusing on their individual effects on muscle mass, strength, or function [23,25]. In contrast, the present review uniquely integrates these two evidence streams by quantitatively synthesizing studies that combined HMB supplementation with structured RT programs in older adults. This approach allows for the evaluation of their potential synergistic effects on MQ and physical performance, thereby addressing a critical gap in the literature and providing a more comprehensive understanding of multimodal interventions targeting sarcopenia.

Focusing on a single, well-defined nutritional strategy constitutes a methodological advantage [27,28]. Given its established role in enhancing recovery after high-intensity exercise [13,24] and its favourable impact on body composition [25], HMB emerges as a promising adjunct to RT. Addressing this critical gap in the existing evidence, the present systematic review and meta-analysis aims to elucidate the specific role of HMB supplementation in modulating and potentially enhancing the effects of RT on body composition, muscle quality, and physical function in adults aged 60 years and older. By doing so, this study contributes to the development of evidence-based strategies to preserve muscle health and functional independence in aging populations. We hypothesized that RT combined with HMB would yield greater improvements in functional outcomes, body composition, and muscle quality compared with RT alone.

## 2. Materials and Methods

### 2.1. Data Sources and Search Strategy

This systematic review was conducted following the recommendations of the Preferred Reporting Items for Systematic Reviews and Meta-Analyses 2020 (PRISMA 2020) guidelines [31,32], and was prospectively registered in the PROSPERO database (registration code: CRD420251144810). A comprehensive literature search was performed in PubMed, Scopus, Cochrane Library, CINAHL, Web of Science (WOS), and ScienceDirect to identify eligible studies from inception to July 2025. The search included original articles published in any language before 21 July 2025. The detailed search strategy is provided in Supplementary Data S1.

### 2.2. Eligibility Criteria

Study screening and eligibility assessment were independently performed by two investigators, with disagreements resolved by consensus through consultation with a third author. Following the PICOS framework (Population, Intervention, Comparison, Outcomes, and Study design) [33], the inclusion criteria were defined as follows:

Population

Studies including older adults aged 60 years or older, of any sex, were eligible for inclusion.

Intervention

Eligible interventions combined RT with HMB supplementation. RT protocols were required to target both upper and lower limbs and could include chair-based programs, weight-bearing resistance exercises, machine-based training, or elastic band exercises. Studies using HMB in either its calcium salt (HMB-Ca) or free acid (HMB-FA) form were considered.

Comparison

Studies were included if they featured control groups performing RT alone, with or without a nutritional placebo supplement.

Outcomes

The outcomes were categorized into three main domains. Physical function outcomes included handgrip strength, gait speed, and SPPB scores. Body composition outcomes comprised appendicular lean mass, fat mass, and body weight. Finally, MQ outcomes encompassed indices or ratios reflecting muscle strength relative to muscle mass. While EWGSOP2 broadly defines muscle quality, operational definitions vary across studies. We extracted MQ data as reported by authors, acknowledging this heterogeneity. MQ was calculated by dividing handgrip strength (kg) by arm muscle mass (kg).

Study design

Only randomized controlled trials (RCTs) and pilot RCTs were included. Studies with no RT, with combined interventions including other supplements, or with non-concurrent administration of HMB and RT (i.e., sequential rather than simultaneous interventions) were excluded.

### 2.3. Study Selection

Study selection was conducted independently by two reviewers (AGA and LPF) using Rayyan software (https://www.rayyan.ai/, accessed on 1 August 2025). Titles and abstracts retrieved from the initial search were screened to exclude irrelevant records, followed by full-text assessment of potentially eligible articles to determine final inclusion. Discrepancies between reviewers were resolved through discussion and, when necessary, consultation with a third reviewer (JLSG). Inter-rater agreement between the two primary reviewers (AGA and LPF) for study selection and quality assessment was evaluated using Cohen’s kappa statistic. Additionally, the reference lists of all included studies were manually screened to identify further eligible publications, and corresponding authors were contacted to obtain missing data or clarifications when required.

### 2.4. Data Extraction

Data were extracted independently by two reviewers using a standardized form. The following information was collected from each study: author, publication year, study design, population characteristics, sample size, age, sex, HMB supplementation and other concurrent supplements, type of RT, intervention volume (duration in weeks, sessions per week, and session length), primary outcome analysed, and compliance. Reported outcomes and main findings were systematically recorded. For both intervention and control groups, pre- and post-intervention means and standard deviations were extracted for quantitative synthesis. When standard deviations (SD) were not provided, they were calculated from the sample size (N) and the standard error (SE) using the formula:

SD = SE × √N.

All outcome measures included in this meta-analysis were assessed using instruments that had been previously validated in the primary studies. Only data reported in those studies were extracted for quantitative synthesis. No additional assessment of the reliability or validation of the instruments was performed for this meta-analysis, as effect sizes were calculated directly from the reported group differences.

### 2.5. Risk of Bias and the Assessment of Methodological Quality of the Studies

The methodological quality of the included studies was evaluated using the Physiotherapy Evidence Database (PEDro) scale [34]. This tool comprises 11 items assessing: eligibility criteria, random allocation, concealed allocation, baseline comparability, blinding of participants, therapists, and assessors, dropout rate (<15%), intention-to-treat analysis, between-group statistical comparisons, and reporting of point measures with variability data. The maximum possible score is 10, as the first item is not included in the total. Scores of 9–10 indicate excellent quality, 6–8 good quality, 4–5 fair quality, and <4 poor methodological quality.

Risk of bias was further assessed using version 2 of the Cochrane Risk of Bias tool for randomized trials (RoB 2) [35]. Each study was evaluated according to the predefined criteria of this tool, which includes six domains: randomization process, deviations from intended interventions, missing outcome data, outcome measurement, selection of the reported result, and overall risk of bias.

### 2.6. Data Synthesis and Analysis

The meta-analysis was conducted using Review Manager software (version 5.4; Cochrane, London, UK). The objective was to examine the effects of HMB supplementation combined with resistance training compared with a control condition (resistance training without HMB or placebo) on muscle quality, physical function (handgrip strength, gait speed, and SPPB score), and body composition (appendicular lean mass, fat mass, and body weight). No a priori sample size or power calculation was performed, as the number of studies included depended on the available literature. However, a post hoc statistical power estimation was carried out for each main outcome domain, considering the number of studies, total sample size, and the magnitude of the pooled effect size.

For each outcome, the standardized mean difference (SMD) and corresponding 95% confidence interval (CI) were calculated based on changes from baseline to post-intervention. The following data were extracted from each study: sample size, mean change, and standard deviation (SD) for both experimental and control groups. When studies reported medians and interquartile ranges instead of means and SDs, these were converted using established methods [36,37]. If only standard errors were reported, they were converted to SDs. When numerical results were unavailable, authors were contacted to obtain the data. If not provided, means and SDs were estimated from graphical representations using Fiji (ImageJ 1.54p) software (National Institutes of Health, Bethesda, MD, USA). When none of these procedures were feasible, the study was excluded from the quantitative synthesis and described narratively.

When pre–post mean differences were not directly reported, they were calculated from the available baseline and post-intervention values. In cases where the SD of the change was missing, it was estimated from other data in the study: (1) using confidence intervals or *p* values, according to the Cochrane Handbook (Section 6.5.2.2) [38]; (2) if unavailable, by applying a correlation coefficient from the most comparable included study (Section 6.5.2.8 of the Cochrane Handbook) [38]; or (3) by assuming a conservative correlation coefficient of 0.5, as previously recommended [39].

Meta-analyses were performed using the inverse variance method under a random-effects model, given the expected variability among studies. Statistical significance was set at *p* < 0.05. Effect sizes were interpreted according to conventional thresholds: SMD ≥ 0.8 (large), 0.5–0.8 (moderate), and 0.2–0.5 (small) [40].

Analyses were only performed for outcomes reported by at least three independent studies; otherwise, the variable was excluded from the pooled analysis.

To evaluate the robustness of the findings, sensitivity analyses were conducted. These included: (1) excluding studies in which SDs were estimated using imputed correlation coefficients (either from another study or the conservative 0.5 value); and (2) performing an outlier analysis, identifying as outliers those studies whose confidence intervals did not overlap with the pooled effect estimate. The meta-analysis was then repeated after excluding these outlier studies to determine whether their removal substantially affected the overall results. Sensitivity analyses were conducted only when a minimum of three studies were available for a given variable.

Heterogeneity was quantified using the *I*^2^ statistic, representing the percentage of total variation across studies due to heterogeneity rather than chance. The following interpretation was used: 0–40% (might not be important), 30–60% (moderate), 50–90% (substantial), and 75–100% (considerable heterogeneity) [41].

Finally, publication bias and potential asymmetry were visually assessed through funnel plots when at least three studies were available for the outcome analysed.

## 3. Results

### 3.1. Search Results and Study Selection

**Figure 1:**
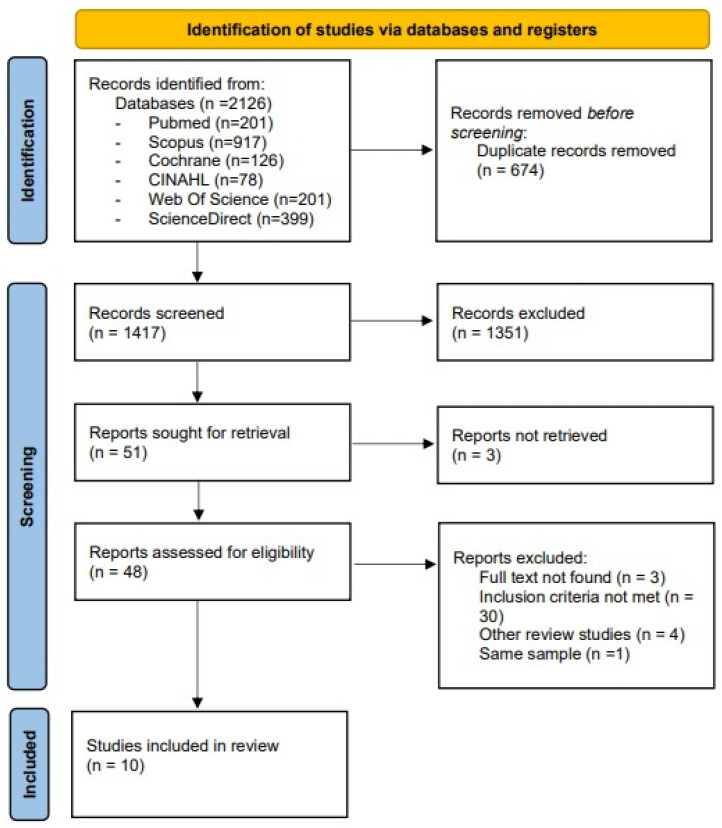
Prisma 2020 Flow Diagram.

The initial search retrieved 2126 records from the PubMed, Scopus, Cochrane Library, CINAHL, Web of Science (WOS), and ScienceDirect databases. After the removal of 674 duplicates, 1417 records remained for title and abstract screening. Of these, 1351 were excluded for not meeting the inclusion criteria. A total of 51 studies were selected for full-text review. Among these, 3 studies could not be retrieved despite attempts to contact the corresponding authors, and 38 were excluded after detailed assessment for not fulfilling the eligibility criteria. Consequently, 10 studies were included in the systematic review. The process of study identification, screening, and inclusion is summarized in the PRISMA flow diagram (Figure 1). The inter-rater reliability between reviewers for study selection was high (κ = 0.85), reflecting almost perfect agreement.

### 3.2. Study Characteristics

A total of 10 studies [42,43,44,45,46,47,48,49,50,51], all of them RCTs except for one pilot RCT [44], were included in the meta-analysis, comprising 596 participants. Sample sizes ranged from 16 to 156 participants. Across the included studies, 206 individuals were allocated to the intervention groups (RT+HMB) and 188 to the control groups (RT). The remaining 197 participants belonged to additional groups described in some studies, such as RT combined with health education, HMB-only supplementation, or non-exercise groups. One study [51] reported five dropouts during follow-up without specifying group allocation.

Participants were older adults aged 60 years and above, with a weighted mean age of 71.2 ± 3.8 years. Women represented the majority of participants (66.3% ± 26.5), although sex distribution varied considerably between studies; one trial included only women [47], while one included only men [50]. Participant characteristics also differed among cohorts: most studies focused on sarcopenic or pre-sarcopenic individuals [42,46,47,49], whereas others enrolled healthy older men [50] or older adults with vitamin D insufficiency [45]. Two studies evaluated participants following rest or immobilization [43,51], and one involved patients awaiting cardiac surgery [48].

The duration of interventions averaged 15.2 ± 14.0 weeks, with two to three weekly sessions in most trials, although one study implemented RT once per week [49]. The RT protocols varied across studies and included chair-based strength training [47], weight-bearing resistance exercises [46], machine-based strength training [44,45], and programs using elastic bands [42,45].

Most studies provided HMB-Ca using 3 g [42,43,44,45,46,51] or 1.5 g [47] per day. One study [48] adjusted the dose according to renal function—3 g/day for participants without kidney impairment and 1.5 g/day for those with renal dysfunction. Only one study [50] administered HMB-FA, and another [49] offered 1.2 g of HMB without specifying the type. None of the 10 included studies reported any serious adverse events associated with HMB supplementation. While one trial documented minor, potentially related complaints—including nausea, stomach discomfort, and increased urinary frequency —the overall incidence of these adverse events was not statistically significant between the HMB group and the placebo group [47]. Additional methodological and intervention details are summarized in Table 1.

### 3.3. Muscle Quality

**Figure 2:**
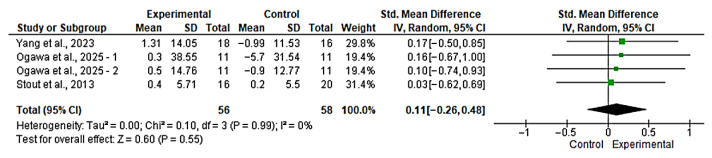
Forest plot for MQ. The analysis includes the following studies: Yang et al., 2023 [42]; Ogawa et al., 2025 [48]; Stout et al., 2013 [44].

Three studies [42,44,48] were included in the evaluation of the effect of the intervention on MQ. Among them, one study [48] included two intervention analyses, one focused on the lower limb and another on the upper limb. The overall analysis showed no significant effect between groups (SMD = 0.11; 95% CI: −0.26 to 0.48; *n* = 114; Z = 0.60; *p* = 0.55), with no evidence of heterogeneity (*p* = 0.99, *I*^2^ = 0%) (Figure 2). The funnel plot showed asymmetry, indicating a possible risk of publication bias (Supplementary Data S2). However, with fewer than 10 studies, formal publication bias assessment is unreliable and these findings should be interpreted with extreme caution. The non-significant SMD (SMD = 0.11) indicates a lack of statistically significant benefit, and the trivial effect size magnitude suggests a lack of clinical relevance for this outcome.

### 3.4. Physical Function

#### 3.4.1. Meta-Analysis for Handgrip Strength

**Figure 3:**
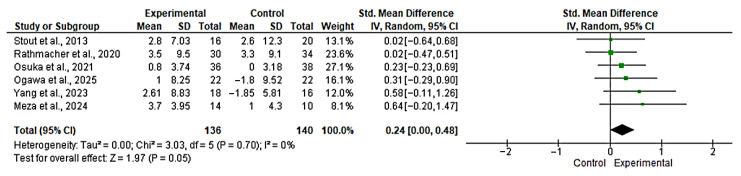
Forest plot for handgrip strength. The analysis includes the following studies: Stout et al., 2013 [44]; Rathmacher et al., 2020 [45]; Osuka et al., 2021 [47]; Ogawa et al., 2025 [48]; Yang et al., 2023 [42]; Meza et al., 2024 [46].

Six studies [42,44,45,46,47,48] were included in the evaluation of the effect of the intervention on handgrip strength. The overall analysis revealed a small but significant effect in favor of the experimental group (SMD = 0.24; 95% CI: 0.0 to 0.48; *n* = 276; Z = 1.96; *p* = 0.05), with no evidence of heterogeneity (*p* = 0.70, *I*^2^ = 0%) (Figure 3). The funnel plot showed asymmetry, indicating a possible risk of publication bias (Supplementary Data S2). However, with fewer than 10 studies, formal publication bias assessment is unreliable and these findings should be interpreted with extreme caution. Based on mean baseline values (~25–30 kg), an SMD of 0.24 corresponds to approximately 1.5–2.0 kg improvement, approaching the MCID of 2–3 kg.

#### 3.4.2. Meta-Analysis for Gait Speed

**Figure 4:**
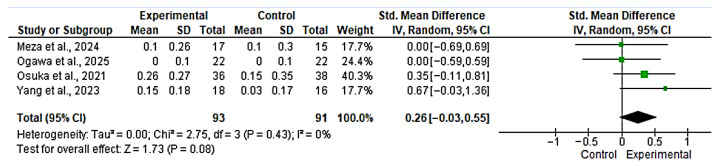
Forest plot for gait speed. The analysis includes the following studies: Meza et al., 2024 [46]; Ogawa et al., 2025 [48]; Yang et al., 2023 [42].

Four studies [42,46,47,48] were included in the evaluation of the effect of the intervention on gait speed. The overall analysis showed a non-significant effect in favor of the experimental group (SMD = 0.26; 95% CI: −0.03 to 0.55; *n* = 184; Z = 1.73; *p* = 0.08), with no evidence of heterogeneity (*p* = 0.43, *I*^2^ = 0%) (Figure 4). The funnel plot showed asymmetry, indicating a possible risk of publication bias (Supplementary Data S2). However, with fewer than 10 studies, formal publication bias assessment is unreliable and these findings should be interpreted with extreme caution. The overall effect was not statistically significant (*p* = 0.08), and the effect size (SMD = 0.26), while small, overlaps with zero, suggesting no clear clinical benefit.

#### 3.4.3. Meta-Analysis for SPPB

**Figure 5:**
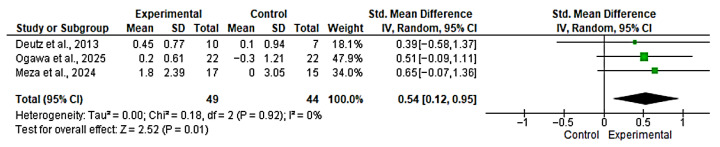
Forest plot for SPPB. The analysis includes the following studies: Deutz et al., 2013 [51]; Ogawa et al., 2025 [48]; Meza et al., 2024 [46].

Three studies [46,48,51] were included in the evaluation of the effect of the intervention on SPB. The overall analysis revealed a significant effect in favor of the experimental group (SMD = 0.54; 95% CI: 0.12 to 0.95; *n* = 93; Z = 2.52; *p* = 0.001), with no evidence of heterogeneity (*p* = 0.92, *I*^2^ = 0%) (Figure 5). A funnel plot was generated to explore potential publication bias; however, with only three studies, the assessment of asymmetry is unreliable (Supplementary Data S2). However, with fewer than 10 studies, formal publication bias assessment is unreliable and these findings should be interpreted with extreme caution. An SMD of 0.54 represents a moderate and clinically relevant benefit, as an increase of 0.5 points on the SPPB scale is considered a minimal significant change, and 1.0 points is considered a substantial change.

### 3.5. Body Composition

#### 3.5.1. Meta-Analysis for Appendicular Lean Mass

**Figure 6:**
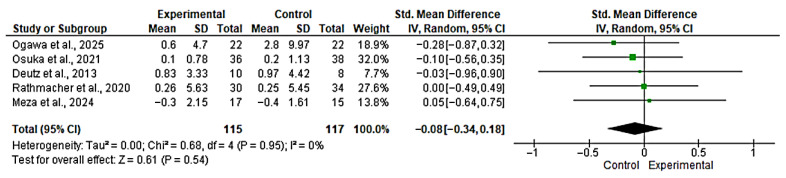
Forest plot for Appendicular Lean Mass. The analysis includes the following studies: Ogawa et al., 2025 [48]; Osuka et al., 2021 [47]; Deutz et al., 2013 [51]; Rathmacher et al., 2020 [45]; Meza et al., 2024 [46].

Five studies [45,46,47,48,51] were included in the evaluation of the effect of the intervention on appendicular lean mass. The overall analysis showed no significant effect between groups (SMD = −0.08; 95% CI: −0.34 to 0.18; *n* = 232; Z = 0.61; *p* = 0.54), with no evidence of heterogeneity (*p* = 0.95, *I*^2^ = 0%) (Figure 6). The funnel plot showed asymmetry, indicating a possible risk of publication bias (Supplementary Data S2). However, with fewer than 10 studies, formal publication bias assessment is unreliable and these findings should be interpreted with extreme caution. The non-significant SMD (*p* > 0.05) and trivial effect size magnitude (SMD < 0.1) suggest a lack of statistical or clinical added benefit from HMB on these body composition variables.

#### 3.5.2. Meta-Analysis for Fat Mass

**Figure 7:**
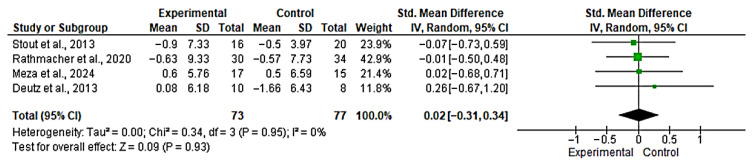
Forest plot for fat mass. The analysis includes the following studies: Stout et al., 2013 [44]; Rathmacher et al., 2020 [45]; Meza et al., 2024 [46]; Deutz et al., 2013 [51].

Four studies [44,45,46,51] were included in the evaluation of the effect of the intervention on fat mass. The overall analysis showed no significant effect between groups (SMD = 0.02; 95% CI: −0.31 to 0.34; *n* = 150; Z = 0.09; *p* = 0.93), with no evidence of heterogeneity (*p* = 0.95, *I*^2^ = 0%) (Figure 7). The funnel plot showed asymmetry, indicating a possible risk of publication bias (Supplementary Data S2). However, with fewer than 10 studies, formal publication bias assessment is unreliable and these findings should be interpreted with extreme caution. The non-significant SMD (*p* > 0.05) and trivial effect size magnitude (SMD < 0.1) suggest a lack of statistical or clinical added benefit from HMB on these body composition variables.

#### 3.5.3. Meta-Analysis for Body Weight

**Figure 8:**
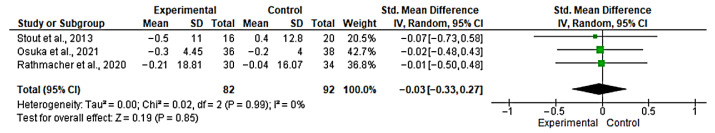
Forest plot for body weight. The analysis includes the following studies: Stout et al., 2013 [44]; Osuka et al., 2021 [47]; Rathmacher et al., 2020 [45].

Three studies [44,45,47] were included in the evaluation of the effect of the intervention on body weight. The overall analysis showed no significant effect between groups (SMD = −0.03; 95% CI: −0.33 to 0.27; *n* = 174; Z = 0.19; *p* = 0.85), with no evidence of heterogeneity (*p* = 0.99, *I*^2^ = 0%) (Figure 8). A funnel plot was generated to explore potential publication bias; however, with only three studies, the assessment of asymmetry is unreliable (Supplementary Data S2). However, with fewer than 10 studies, formal publication bias assessment is unreliable and these findings should be interpreted with extreme caution. The non-significant SMD (*p* > 0.05) and trivial effect size magnitude (SMD < 0.1) suggest a lack of statistical or clinical added benefit from HMB on these body composition variables.

### 3.6. Risk of Bias

The risk of bias assessment was performed using the Cochrane Rob 2 tool for randomized parallel trials for all studies. Overall, a significant portion of the included studies presented methodological concerns. Specifically, five studies [43,45,48,49,51] were judged to be at a high risk of bias, three [44,47,50] raised some concerns and only two [42,46] were determined to have a low risk of bias across all domains.

**Figure 9:**
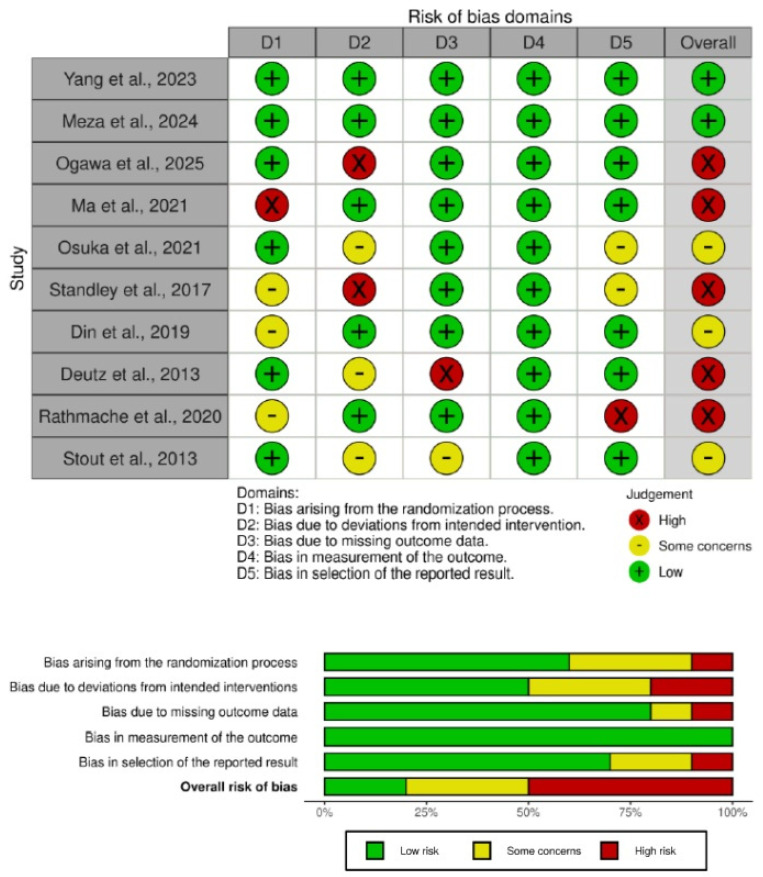
Risk of bias assessment for the included studies. D1–D5 represent bias from randomization, deviations from intervention, missing data, outcome measurement, and reporting. Judgments are classified qualitatively as Low (green), Some concerns (yellow), or High (red). No numerical score was applied, as the RoB 2 tool uses categorical rather than quantitative evaluation. The analysis includes the following studies: Yang et al., 2023 [42]; Meza et al., 2024 [46]; Ogawa et al., 2025 [48]; Ma et al., 2021 [49]; Osuka et al., 2021 [47]; Standley et al., 2017 [43]; Din et al., 2019 [50]; Deutz et al., 2013 [51]; Rathmacher et al., 2020 [45]; Stout et al., 2013 [44].

The primary drivers for the high risk of bias rating were issues identified in the domains of bias arising from the randomization process (D1), bias due to deviations from the intended interventions (D2) and bias in the selection of the reported result (D5). Similarly, the “some concerns” ratings were most frequently attributed to a lack of clarity in the randomization process and deviations from the intended interventions. A summary of the overall assessments is presented in Figure 9.

### 3.7. Methodological Quality

Across the ten included studies, PEDro scores ranged from 6 to 10, with a mean score of 8.0, indicating overall good methodological quality. Two studies achieved the maximum score of 10 points [42,46], one study scored 9 points [51], three studies scored 8 points [47,48,50], one study scored 7 points [43], and three studies scored 6 points [44,45,49]. Most studies demonstrated a robust design, fulfilling key methodological criteria such as random allocation, baseline group equivalence, and assessor blinding. The main limitation identified was the lack of therapist blinding (criterion 6 of the PEDro scale), a common constraint in non-pharmacological intervention research that may introduce some risk of bias. Additionally, a few studies did not clearly describe the allocation concealment procedure. Detailed PEDro scores for each study are presented in Table 2.

### 3.8. Post Hoc Power Analysis

For this meta-analysis, no a priori sample size or power calculation was performed, as the number of studies included was determined by the available literature. However, a post hoc estimation of statistical power was conducted for each main outcome domain based on the number of studies, total sample size, and magnitude of the observed pooled effect size.

For the physical function domain, which included analyses of handgrip strength (k = 6; *n* = 276; SMD = 0.24), gait speed (k = 4; *n* = 184; SMD = 0.26), and SPPB (k = 3; *n* = 93; SMD = 0.54), the estimated power ranged from approximately 40% to 75%, depending on the number of studies and effect magnitude. For the body composition domain, including appendicular lean mass (k = 5; *n* = 232; SMD = −0.08), fat mass (k = 4; *n* = 150; SMD = 0.02), and body weight (k = 3; *n* = 174; SMD = −0.03), the estimated power was below 40% to detect small-to-moderate effects (Cohen’s d = 0.3–0.5) with α = 0.05.

Overall, these estimations suggest that the meta-analyses had sufficient statistical power to detect moderate-to-large effects in some functional outcomes (e.g., SPPB) but were likely underpowered to detect small effects, particularly in the body composition domain. Consequently, non-significant results should be interpreted with caution, as they may reflect limited statistical power rather than a true absence of effect.

## 4. Discussion

To our knowledge, this is the first systematic review and meta-analysis to determine the effects of RT combined with HMB simultaneously on MQ, body composition and physical function in older adults. Based on the studies included in the meta-analysis, we found that HMB supplementation combined with RT modestly improved muscle strength and physical performance but did not significantly affect muscle quality or body composition.

The handgrip strength result (SMD = 0.24, 95% CI: 0.00–0.48, *p* = 0.05) represents borderline significance with considerable uncertainty. While reaching conventional significance threshold, replication in larger trials is needed. The finding of improved function and handgrip strength is in line with the recommendations of the EWGSOP2 [10]. This consensus emphasises that low muscle strength is the defining parameter of sarcopenia, relegating muscle mass and physical performance to second and third place, respectively. The conceptual definition of sarcopenia by the GLIS [12] also includes muscle strength, although the next steps for the expert group will be to translate this definition into an operational definition that allows the use of common terminology, measures, and cut-off points. For this reason, the use of HMB alongside an RT programme should be considered to prevent loss of function and strength in older adults, as well as in the prevention of sarcopenia. It is important to note that within the SPPB, the repeated chair-stand test is the most strength-dependent and training-responsive component. This subtest has consistently demonstrated higher sensitivity to RT–induced changes than gait speed or balance tasks, and is strongly associated with mobility limitations, disability, and mortality in older adults. Therefore, our interpretation of SPPB improvements places particular emphasis on changes in chair-stand performance, which likely represent the main driver of the observed functional gains.

No significant effects were observed on gait speed, appendicular muscle mass, MQ, fat mass or body weight. The absence of a significant effect of HMB on body composition may be attributed to the potent anabolic stimulus conferred by RT itself. Indeed, RT alone has been shown to elicit favourable changes in body composition in older adults with sarcopenic obesity when compared with non-exercising controls [23]. Recent findings suggest that power training may further amplify this anabolic response, leading to greater increases in muscle mass and reductions in fat within this population [52]. Similar improvements have also been observed in cancer survivors following treatment [53] and in healthy adults [54], particularly reflected in reductions in body fat. Moreover, RT is more effective at increasing lean body mass than aerobic exercise in this population [55].

It is therefore plausible that, within the context of a well-structured RT program, the incremental effect of HMB is modest and may be masked by the dominant training stimulus, unlike in meta-analyses including heterogeneous populations subject to less controlled or variable exercise regimens [26,29] or instance, Lin et al. concluded that HMB supplementation could increase muscle mass but found no effect on fat mass. For this reason, when reviewing the literature, it is essential to distinguish between studies that compare RT+HMB versus RT alone, those combining HMB with general exercise regimens, or those combining RT with other nutritional interventions. Only by parsing these designs can one more clearly isolate the unique contribution of HMB beyond the powerful effects of RT. Furthermore, future research should aim to compare different subtypes of RT (e.g., traditional strength training, power-oriented training, free-weight protocols, or elastic band exercises) to determine which modality may optimise the ergogenic effects of HMB. It would also be advisable to consider the duration of the interventions, as studies lasting fewer than 12 weeks may not provide sufficient time for measurable and meaningful adaptations to occur in the assessed variables.

Of particular interest is the trend toward improvement in MQ observed in the group receiving HMB supplementation. Although this difference did not reach statistical significance, likely due to the heterogeneity in assessment methods and the lack of a standardised definition, these findings suggest that the combination of RT and HMB may exert a beneficial effect that was not fully captured in our study. The heterogeneity in MQ evaluation across studies further complicates interpretation. For example, Stout et al. [44] assessed MQ in both upper limbs, whereas other two studies [42,48] evaluated it in a single arm or leg, introducing variability that may influence the magnitude and reliability of the reported effects. This diversity in measurement approaches reflects the broader lack of consensus [12] on how MQ should be defined and quantified. Future research should therefore include larger and more homogeneous samples to clarify whether this potential benefit is genuine and clinically meaningful. In parallel, the establishment of a standardised and widely accepted definition of MQ is essential to improve comparability across studies and facilitate the integration of findings into clinical and functional contexts.

One of the main strengths of our study is the inclusion of studies that only used RT, which is a well-established and relevant type of exercise in clinical practice. Furthermore, the use of HMB as a single supplement allows its effect to be isolated, unlike other studies that used different nutritional supplements. Another relevant limitation is that none of the included studies evaluated participants’ nutritional status or baseline dietary intake. This omission is important because HMB efficacy may be modulated by baseline nutritional status, such as total protein intake, dietary leucine consumption, or vitamin D levels. If baseline protein intake is already optimal, the additive effect of HMB may be negligible. Future studies should control for these variables to better isolate the effects of HMB [56].

This study presents several limitations. Although ten studies were included in the systematic review, only seven could be incorporated into the meta-analysis due to the lack of comparable quantitative data or heterogeneity in the reported outcomes. The diversity of study populations and methodologies may also limit the generalisability of our findings. It should be noted that, in the analysis of handgrip strength, one study [46] included data from women only, as the standard deviation was reported exclusively for the female subgroup. Consequently, male participants from that study could not be incorporated into the quantitative synthesis. This methodological detail may have slightly influenced the overall pooled estimate, given the potential sex-related differences in muscle strength and response to RT and nutritional supplementation.

Another limitation is that the review was limited to adults aged 60 and over; therefore, the findings cannot be generalized to younger or middle-aged populations. On the other hand, despite the fact that all the measurement instruments used in the studies were validated, there is heterogeneity in measurements as there is no consensus. The duration of interventions was variable, and some [48,51] (e.g., <12 weeks) may have been too short to elicit significant changes in body composition, which also precluded a dose–response analysis. Finally, the consistently low heterogeneity may reflect small study numbers limiting power to detect heterogeneity, or genuine consistency across similar study designs. Exploratory subgroup analyses were not performed because the number of available trials per outcome was too small to support reliable stratification. Conducting such analyses under sparse-data conditions would risk unstable or misleading estimates. Future RCTs with larger and more homogeneous samples are needed to allow meaningful subgroup exploration. It is also important to distinguish between studies comparing RT+HMB with a nutritional placebo and those comparing RT+HMB with RT alone, as the observed effects may differ depending on the control condition. Two trials [48,49] did not include a placebo group, which limits the ability to isolate the specific effect of HMB from that of RT itself. The small number of male participants also limits the generalisability of these findings. Future trials with larger, more homogeneous samples are needed to confirm these trends and clarify their clinical relevance. From a practical perspective, RT should remain the cornerstone of interventions targeting muscle function, with HMB potentially serving as a useful adjunct to optimise strength outcomes.

It would be essential to conduct longer-term RCTs with larger samples to confirm the observed trends and determine whether the effects of HMB on body composition emerge in the long term. Although SUCRA (Surface Under the Cumulative Ranking Curve) is frequently used in network meta-analyses to rank multiple interventions, it does not apply to our review because we performed a pairwise comparison between RT+HMB and RT alone. In simple terms, SUCRA expresses the probability that a treatment is among the most effective options (0% to 100%). This brief explanation is provided to aid non-specialist readers, while clarifying that such ranking methods were not required in our analysis.

In summary, this meta-analysis provides evidence that HMB supplementation combined with RT modestly enhances functional performance in older adults, particularly muscle strength and SPPB outcomes. Although effects on muscle mass and MQ were limited, these findings highlight the potential of HMB as a safe and accessible adjunct to RT in the prevention of age-related functional decline. The modest magnitude of improvement underscores that RT remains the primary driver of musculoskeletal adaptation, while HMB may offer an incremental advantage, particularly in populations with diminished anabolic responsiveness or inadequate dietary protein intake. From a translational perspective, the present findings support the integration of combined RT and HMB strategies into multidisciplinary programs for the prevention of sarcopenia and frailty. Nevertheless, the heterogeneity of existing evidence underscores the need for harmonized protocols and standardized assessments of muscle quality to facilitate comparison across studies. Future research should also examine the interaction between nutritional supplementation and different RT modalities (e.g., power training, elastic resistance, or functional exercise), as well as explore dose–response relationships and long-term safety outcomes. Future research should prioritise conducting long-term, adequately powered RCTs that compare RT+HMB with well-matched RT-only or placebo-controlled conditions; developing standardised, consensus-based protocols for assessing muscle quality and functional performance to reduce heterogeneity across studies; systematically evaluating baseline nutritional status—including protein intake, leucine exposure and vitamin D levels—to identify individuals who may benefit most from HMB; and examining the potential interactions between HMB supplementation and specific resistance training modalities, such as high-velocity or elastic-resistance exercise, which may induce distinct neuromuscular and metabolic adaptations. Addressing these priorities will strengthen the evidence base and help define the true clinical relevance of combining RT with HMB in ageing populations.

## 5. Conclusions

This systematic review and meta-analysis indicates that HMB supplementation may modestly enhance functional outcomes when combined with RT, although its effects on muscle mass and quality remain uncertain. The observed improvements, particularly in handgrip strength and SPPB performance, suggest a potential synergistic effect when HMB is used alongside RT. However, no consistent benefits were found for body composition or MQ, possibly due to the potent anabolic and neuromuscular stimulus of RT itself or to the relatively short duration of several interventions, which may have limited the detection of measurable structural changes. Overall, RT should remain the cornerstone of interventions aimed at preserving muscle strength and independence in aging populations, with HMB serving as a safe, well-tolerated, and potentially useful adjunct. Further large-scale, long-duration RCTs employing standardized MQ assessments are warranted to clarify the long-term clinical relevance of RT combined with HMB in promoting healthy aging and functional independence.

## Figures and Tables

**Table 1:** Baseline Characteristics.

Author (Year)	Type of Study	Population	Total *n*	Age (Years)	Sex (%)	HMB Supplementation and Other Supplementation	Type of Resistance Training	Volume			Main Variables Analysed	Compliance
								Weeks	Sessions per Week	Duration		
Yang, 2023 [42]	RCT	≥60a sarcopenic (AWGS)	34. HMB (18) and CG (16)	HMB (72.9) and CG (71.4)	Women: HMB 61%, CG 68.8% (total 64.7%). Men: HMB 39%, CG 31.2% (total 35.3%).	3 g product: 1500 mg HMB-Ca, 20 kJ energy, 0 g protein/fat, 1.2 g carbs, 15 mg sodium. HMB or placebo twice/day.	CG and HMB groups performed resistance exercises with yellow elastic bands (3 lb), including 4 upper- and 5 lower-limb movements, completed in 2 sets of 5–8 repetitions.	12	2	40′	Hand grip strength Gait speed Five time chair stand test SMM SMI FFM SLM FFM of the right arm MQ TWEAK IL-18 Fasting blood glucose Total cholesterol Triglycerides LDL cholesterol HDL cholesterol	Supplementation: individual compliance with HMB or placebo was monitored by asking subjects to return the empty sachets. RT: Both the HMB and CG received the supervised RT.
Din, 2019 [50]	RCT	Healthy older men	16. HMB-FA (8) y CG (8)	HMB-FA (67.8) CG (68.5)	100% men	Each HMB-FA packet contained 1 g of HMB-free acid, Litesse polydextrose, reverse osmosis water, bitter-reducing agent, orange flavor, stevia extract, citric acid, potassium sorbate, and powdered xanthine gum. The supplements were plain-wrapped. 3 packets per day.	Both groups performed supervised unilateral RET (dominant leg extension), 6 sets of 8 repetitions at 75% of 1 RM	6	3	≈15–25′	MVC (maximal voluntary contraction) 1-RM Thigh lean mass Thigh fat free mass VL thickness Pennation angle Fibre length Plasma HMB concentration D2O-derived muscle protein synthesis (MPS) Expression of genes involved in muscle hypertrophy/atrophy and myogenesis.	Supplementation: a log of returned sachetswas kept to inform upon compliance, whichwas excellent in both groups (100% PLA vs. 99% HMB). RT: was supervised
Rathmacher, 2020 [45]	RCT	≥60, 25OH-D insufficiency (15–30 ng/mL)	117. HMB + D (no ex) 27 CG (no ex) 26 HMB + D (ex) 30 CG (ex) 34	HMB + D (no ex) 71 CG (no ex) 70.8 HMB + D (ex) 67.2 CG (ex) 67.7	HMB + D (no ex) W (55.5%) M (44.4%) CG (no ex) W (69.2%) M (30.8%) HMB + D (ex) W (53.3%) M (46.7%) Control (EX) W (64.7%) M (35.3%)	HMB + D: Ca-HMB (3 g per day) and vitamin D (2000 IU per day) both divided into 2 doses (morning and afternoon). Both supplements contained calcium (102 mg), phosphorus (26 mg) and potassium (49 mg). Placebo: calcium lactate.	Progressive resistance training program including 11 upper- and lower-body exercises; three sets per exercise (two sets of up to 15 repetitions and one set of up to 20 repetitions), progressing from elastic bands to weight machines as muscular strength improved.	52	3	60′	Weight BMI (kg/m^2^) Lean mass (kg) Muscle mass Fat mass (kg) Body fat (%) Trunk lean mas trunk fat mass appendicular fat mass (kg) appendicular lean mass (kg) Bone mineral density (g/cm^3^) Muscle strength (bilateral knee and elbow) Get up (reps) Get up and go (s) Grip strength (kg) Dietary assessment Blood and orine sample SF-36 Circumplex Affect questionnaire	Supplementation: Mean ± SE ingestion of supplements was 96.4% ± 0.7% in the exercise + HMB + D group, 96.0% ± 0.7% in the control group, RT: Mean ± SD attendance at the exercise programs was 83.2% ± 2.2% in the exercise + HMB + D group, 83.4% ± 2.0% in the control group.
Meza, 2024 [46]	RCT	Men and women aged 60 years or older who had been discharged from a post-acute geriatric rehabilitation unit within the previous 3 months, diagnosed with sarcopenia according to EWGSOP2 criteria.	32. HMB (17) CG (15)	81.6. HMB (81.8) CG (81.3)	75% women and 25% men. HMB W (82.4%) M (17.6%) CG W (66.7%) M (33.3%)	The HMBG received a dose of 3 g/day of Ca-HMB. The CG received the same amount of placebo in the form of maltodextrin.	Both the HMBG and CG performed resistance training targeting the upper and lower extremities, specifically the shoulder flexors and abductors, the elbow flexors, the hip flexors and abductors, the knee flexors and extensors, and the plantar flexors. The initial load was 0.5 kg, and the total weight was 3 kg for each extremity by the end of the intervention.	12	3	60′	BMI Handgrip strength (kg) Gait speed (m/s) SPPB Fat-free mass (FFM) with BIA (kg) Fat-free mass index (kg/m^2^) Fat mass (kg) Fat mass index (kg/m^2^) Activity capacity (Barthel index and Lawton scale) SarQol Charlson Comorbidity Index	Supplementation: adherence to the prescribed intake of packets was high in both groups (*p* = 0.589). RT: Of the total participants, 21 individuals (65.6%) completed 70% or more of the exercise sessions, comprising 13 participants from the intervention group and eight from the control group (*p* = 0.266).
Osuka, 2021 [47]	RCT	Women ≥65 y, low muscle mass (AWGS)	156. RT +HMB: 39 RT + placebo: 39 Health education + HMB: 39 Health education + placebo:39	72.1. RT +HMB: 73.5 RT + placebo: 71.8 Health education + HMB: 71.5 Health education + placebo: 71.6	100% women	The Ca-HMB supplement contained 3.5 g carbohydrates, 30 mg protein, 20 mg fat, 0.2 mg sodium, 207 mg calcium, and 1200 mg HMB. Participants dissolved 4.2 g of powder in 200 mL water, consumed it once daily after a meal, and recorded intake in a diary. The placebo, matched in weight, replaced calcium and HMB with 0.6 g carbohydrates and maltitol	Chair strength training during weeks 1–12, resistance band strength training during weeks 5–7, ankle weight strength training during weeks 7–12, and machine strength training during weeks 9–12.	12	2	60′	Weight BMI Lean mass (kg) Lean mass (kg) Appendicular lean mass (kg) Fat-free mass (kg) Skeletal muscle index (kg/m^2^) Knee extensor strength Hip adductor strength Grip strength (kg) Usual gait speed (m/s) Maximum gait speed (m/s) Timed start and walk time 5-rep sit-to-stand exercise TMIG-IC (score) Blood markers Usual dietary intake Usual physical activity levels	Supplementation: Mean ± SD ingestion of supplements was 91.6% ± 21.2% in the exercise + HMB group, 87.1% ± 27.8% in the exercise+placebo group. RT: Mean ± SD attendance at the exercise programs was 95.2% ± 4.8% in the exercise + HMB group, 92.9% ± 15.4% in the exercise + placebo group.
Stout, 2013 [44]	RCT	≥65 y, Geriatric Nutritional Risk Index ≥92, BMI >20 <30, ambulatory	108. NE+PL: 27 NE+HMB: 27 RT+PL: 27 RT+HMB: 27	NE+PL: 72 NE+HMB: 73 RT+PL: 73 RT+HMB: 73	NE+PL: M 56% W 44% NE+HMB: M 52% W 48% RT+PL: M 54.2% W 45.8% RT+HMB: M 54.2% W 45.8%	3 g/day Ca-HMB (2 × 1.5 g sachets + carbs)	RT volume progressed throughout the intervention, beginning with a pre-test in week 1. Participants performed 1 set per exercise in weeks 2–3, 2 sets in week 4, and 3 sets in weeks 5–10. Week 11 involved 1–2 sets, followed by a mid-test in week 12. Weeks 13–14 included 1 set, week 15 2 sets, weeks 16–22 3 sets, and week 23 1–2 sets, concluding with the post-test in week 24. Exercises consisted of bench press, lat pulldown, bilateral leg press, hack squat, and bilateral leg extension, with 1–3 sets of 8–12 repetitions per exercise.	24	3	≈40–60′	Weight Total lean mass (kg) Leg lean mass (kg) Total fat mass Extensor isokinetic peak torque 60° (Nm) Flexor isokinetic peak torque 60° (Nm) Extensor isokinetic peak torque 180° (Nm) Flexor isokinetic peak torque 180° (Nm) Grip strength (kg) Muscle quality 60° Nm/kg Muscle quality 180° Nm/kg Grip strength muscle quality Get up and go (s) Activities of daily living	Supplementation: product intake was recorded on individual intake logs, which were returned to the laboratory and monitored. Urinary HMB levels were used as markers to indicate test treatment compliance. Minimum adherence: consumed >67% of study product; and completed >60% of RE sessions RT: All RE sessions were completed in the laboratory
Deutz, 2013 [51]	RCT	Older adults (60–79 y), healthy (SPPB ≥9), 10 days bed rest	24 (20 women y 4 men). CG (8) HMB (11)	CG (67.1) HMB 67.4)	24 (20 women y 4 men). CG: W 87.5% M 12.5 HMB W 72.73 M 27.27	Each HMB sachet contained 1.5 g Ca-HMB, 4 g maltodextrin, 200 mg calcium, and additional sweeteners and flavoring. The control sachet had the same composition, excluding Ca-HMB. Two sachets were administered daily, starting five days before a 10-day bed rest period and continuing through the rehabilitation phase.	RT involved circuit exercises targeting hip and knee extensors and flexors, along with light upper-body exercises. Participants performed three sets of 8–10 repetitions at ~80% of 1 RM, with 2 s concentric and 4–6 s eccentric contractions, and appropriate rest between sets. The 1 RM was assessed weekly	8	3	60′	Weight Total body fat (kg) Bone mineral density (g/cm^2^) Fasting blood glucose (mg/dL) Total cholesterol (mg/dL) Serum albumin (g/dL) CRP (mg/L) SPPB score Total lean mass (kg) Appendicular lean mass (kg) Leg lean mass (kg) Upper arm lean mass (kg) Trunk lean mass (kg) Total body fat mass (kg) Leg fat mass (kg) Upper arm fat mass (kg) Trunk fat mass (kg)	Supplementation: Not evaluable if the subject had <67% of total study product consumption at final visit/exit as determined by product consumption records.
Ma, 2021 [49]	RCT	≥65 y, sarcopenic (AWGS)	46. EG: 11 EHMB: 23 CG:12	EG: 76.4 EHMB: 73.7 CG:69.3	EG: W 54.5% M 45.5% EHMB: W: 52.2% M 47.8% CG:W 50% M 50%	2 sachets daily. Each sachet (54.1 g of powder) contained 231 calories, 8.61 g of protein, 1.21 g of β-hydroxy β-methylbutyrate, 130 IU of vitamin D, and 0.29 g of omega-3 fatty acid.	Resistance exercises were performed for 20 to 30 min and aerobic exercises for 20 min weekly.	12	1	50′	Genetic expression: RASGRP1 BIN1 LEF1 ANXA6 IL-7R LRRN3 PRKCQ	
Standley, 2017 [43]	RCT	Older adults (60–76 y)	19. IG (11) CG (8)	IG (67) CG (67)	CG: W 72.73 M 27.27 IG W: 87.5 M: 12.5	Ca-HMB 3 g/day	Both groups, after a 10-day bed rest period, underwent 8 weeks of progressive resistance training (RT) rehabilitation of the upper and lower extremities.	10	3	60′	Skeletal muscle cross-sectional area Mitochondrial content Mitochondrial dynamics and muscle proteolysis lipidomics	
Ogawa, 2025 [48]	RCT	≥65 y, cardiac surgery patients, ≥2 wks nutritional supplementation feasible	44. HMB 22 CG 22	HMB (71.8) CG (73.3)	HMB W 32% M 68% CG W (41%) M (59%)	IG received HMB supplementation for at least 14 days prior to surgery. Each Abound^®^ packet (24 g) contained 1500 mg of HMB-Ca, 7000 mg of L-glutamine, 7000 mg of L-arginine, no protein, and provided 79 kcal of energy. Patients without chronic kidney disease received one packet twice daily, whereas those with chronic kidney disease (eGFR < 60 mL/min/1.73 m^2^) received one packet daily. The control group did not receive any nutritional support.	In both groups, patients received instructions from a physical therapist regarding exercise during the waiting period before surgery. Specifically, RT were prescribed for each patient according to their abilities.	At least 2 with an average of between 18 and 39 days.		All evaluations were conducted at baseline (T1), on the day before surgery (T2), and 2 weeks after surgery (T3). HMB supplementation was taken between T1 and T2	6MWT (m) Grip strength Knee extensor muscle strength SPPB Gait speed Appendicular skeletal muscle mass Upper limb muscle quality Lower limb muscle quality Phase angle (°) Length of hospital stay and incidence of complications	Supplementation: Patients with a compliance rate of <50% were considered to be dropped out. Treatment adherence rate in the HMB group was 95.2% (range, 77.3–100.0%), and no patients dropped out of taking supplements.

Abbreviations: HMB, β-hydroxy-β-methylbutyrate; RCT, randomized controlled trial; AWGS, Asian Working Group for Sarcopenia 2019; HMB, β-hydroxy-β-methylbutyrate group; CG, control group; HMB-Ca, calcium β-hydroxy-β-methylbutyrate; RT, resistance training; RET, resistance exercise training; SMM, skeletal muscle mass; SMI, skeletal muscle index; FFM, fat free mass; SLM, soft lean mass; MQ, muscle quality; TWEAK, Tumor necrosis factor-like weak inducer of apoptosis; HMB-FA, β-hydroxy-β-methylbutyrate free acid; 1 RM, one repetition maximum; MVC, maximal voluntary contraction; VL, vastus lateralis; MPS, muscle protein synthesis; D2O, deuterium oxide; PLA, placebo; 25OH-D, 25-hydroxyvitamin D; EX, exercise; W, women; M, men; BMI, body mass index; SF-36, Short Form Health Survey 36; SD, standard deviation; IG, intervention group; IU, international unit; SPPB, Short Physical Performance Battery; FFM, fat-free mass; BIA, bioelectrical impedance analysis; SarQol, Sarcopenia and Quality of Life questionnaire; TMIG-IC, Tokyo Metropolitan Institute of Gerontology Index of Competence; NE, no exercise; PL, placebo; Nm, Newton meter; CRP, C-reactive protein; EG, exercise group; EHMB, exercise + β-hydroxy-β-methylbutyrate group; RASGRP1, RAS guanyl releasing protein 1; BIN1, bridging integrator 1; LEF1, lymphoid enhancer-binding factor 1; ANXA6, annexin A6; IL-7R, interleukin-7 receptor; LRRN3, leucine-rich repeat neuronal 3; PRKCQ, protein kinase C theta; eGFR, estimated glomerular filtration rate; 6MWT, six-minute walk test.

**Table 2:** Methodological score of RCTs using the PEDro scale.

Study	1	2	3	4	5	6	7	8	9	10	11	Total
Yang et al., 2023 [42]	Y	Y	Y	Y	Y	Y	Y	Y	Y	Y	Y	10
Deutz et al., 2013 [51]	Y	Y	Y	Y	Y	Y	Y	Y	N	Y	Y	9
Din et al., 2019 [50]	Y	Y	N	Y	Y	N	Y	Y	Y	Y	Y	8
Ma et al., 2021 [49]	N	Y	N	Y	N	N	N	Y	Y	Y	Y	6
Meza et al., 2024 [46]	Y	Y	Y	Y	Y	Y	Y	Y	Y	Y	Y	10
Ogawa et al., 2025 [48]	Y	Y	Y	Y	N	N	Y	Y	Y	Y	Y	8
Osuka et al., 2021 [47]	Y	Y	Y	Y	Y	N	Y	Y	N	Y	Y	8
Rathmacher et al., 2020 [45]	Y	Y	N	Y	Y	N	Y	N	N	Y	Y	6
Standley et al., 2017 [43]	Y	Y	N	Y	Y	N	N	Y	Y	Y	Y	7
Stout et al., 2013 [44]	Y	Y	N	Y	Y	N	Y	N	N	Y	Y	6

## Data Availability

No datasets were generated or analysed during the current study.

## Supplementary Materials

The following supporting information can be downloaded at: https://www.mdpi.com/article/10.3390/nu17223624/s1: Supplementary Data S1: Search strategy used in electronic databases; Supplementary Data S2: Funnel plots for publication bias assessment.

## Abbreviations

The following abbreviations are used in this manuscript:

Abbreviation	Meaning
BMI	Body Mass Index
CI	Confidence Interval
EWGSOP2	European Working Group on Sarcopenia in Older People 2
GLIS	Global Leadership Initiative on Sarcopenia
HMB	β-Hydroxy-β-Methylbutyrate
HMB-Ca	Calcium β-Hydroxy-β-Methylbutyrate
HMB-FA	Free Acid β-Hydroxy-β-Methylbutyrate
IGF-1	Insulin-Like Growth Factor 1
MQ	Muscle Quality
PEDro	Physiotherapy Evidence Database
PICOS	Population, Intervention, Comparison, Outcomes, and Study Design
PRISMA	Preferred Reporting Items for Systematic Reviews and Meta-Analyses
PROSPERO	International Prospective Register of Systematic Reviews
RCT	Randomized Controlled Trial
RoB 2	Cochrane Risk of Bias 2 Tool
RT	Resistance Training
SD	Standard Deviation
SE	Standard Error
SMD	Standardized Mean Difference
SPPB	Short Physical Performance Battery
WOS	Web of Science
