# Supplementary material for: The Role of HMB Supplementation in Enhancing the Effects of Resistance Training in Older Adults: A Systematic Review and Meta-Analysis on Muscle Quality, Body Composition, and Physical Function
Source: Nutrients. 2025 Nov 20;17(22):3624. doi: 10.3390/nu17223624 (PMC12655442; doi:10.3390/nu17223624)
Supplement: Supplementary file 1 [file nutrients-17-03624-s001.zip › Supplementary Data 1.pdf]

Supplementary Data 1. The retriveal strategy

Pubmed Search Formula: 201

(HMB OR "HMB Supplementation" OR "Hydroxy-beta-methylbutyrate" OR "Hydroxy-beta-methylbutyrate supplementation" OR "β-Hydroxy β-Methylbutyrate" OR "Beta-Hydroxy Beta-Methylbutyrate" OR "Ca-HMB" OR "FA-HMB" OR "Calcium HMB" OR "HMB free acid" OR "Beta-hidroxi-beta-metilbutirato") AND ("Resistance Training" OR "Strength Training" OR "Progressive Resistance Training" OR "Strength Exercise" OR "Resistance Exercise" OR "Resistance Strength Training" OR "Power Training" OR "Physical Exercise" OR "Weight Training" OR "Force Training" OR "Fitness Training" OR "Impact Exercise" OR "Elastic Resistance" OR "Elastic Exercise" OR "Loading" OR "Progressive" OR "High Speed")

Scopus Search Formula: 917

TITLE-ABS-KEY(HMB OR "HMB Supplementation" OR "Hydroxy-beta-methylbutyrate" OR "Hydroxy-beta-methylbutyrate supplementation" OR "β-Hydroxy β-Methylbutyrate" OR "Beta-Hydroxy Beta-Methylbutyrate" OR "Ca-HMB" OR "FA-HMB" OR "Calcium HMB" OR "HMB free acid" OR "Beta-hidroxi-beta-metilbutirato") AND ("Resistance Training" OR "Strength Training" OR "Progressive Resistance Training" OR "Strength Exercise" OR "Resistance Exercise" OR "Resistance Strength Training" OR "Power Training" OR "Physical Exercise" OR "Weight Training" OR "Force Training" OR "Fitness Training" OR "Impact Exercise" OR "Elastic Resistance" OR "Elastic Exercise" OR "Loading" OR "Progressive" OR "High Speed")

Cochrane Library Search Formula: 126

(HMB OR "HMB Supplementation" OR "Hydroxy-beta-methylbutyrate" OR "Hydroxy-beta-methylbutyrate supplementation" OR "β-Hydroxy β-Methylbutyrate" OR "Beta-Hydroxy Beta-Methylbutyrate" OR "Ca-HMB" OR "FA-HMB" OR "Calcium HMB" OR "HMB free acid" OR "Beta-hidroxi-beta-metilbutirato") AND ("Resistance Training" OR "Strength Training" OR "Progressive Resistance Training" OR "Strength Exercise" OR "Resistance Exercise" OR "Resistance Strength Training" OR "Power Training" OR "Physical Exercise" OR "Weight Training" OR "Force Training" OR "Fitness Training" OR "Impact Exercise" OR "Elastic Resistance" OR "Elastic Exercise" OR "Loading" OR "Progressive" OR "High Speed")

Cinahl Search Formula: 78

(HMB OR "HMB Supplementation" OR "Hydroxy-beta-methylbutyrate" OR "Hydroxy-beta-methylbutyrate supplementation" OR "β-Hydroxy β-Methylbutyrate" OR "Beta-Hydroxy Beta-Methylbutyrate" OR "Ca-HMB" OR "FA-HMB" OR "Calcium HMB" OR "HMB free acid" OR "Beta-hidroxi-beta-metilbutirato") AND ("Resistance Training" OR "Strength Training" OR "Progressive Resistance Training" OR "Strength Exercise" OR "Resistance Exercise" OR "Resistance Strength Training" OR "Power Training" OR "Physical Exercise" OR "Weight Training" OR "Force Training" OR "Fitness Training" OR "Impact Exercise" OR "Elastic Resistance" OR "Elastic Exercise" OR "Loading" OR "Progressive" OR "High Speed")

Web of Science (WOS) Search Formula: 201

(HMB OR "HMB Supplementation" OR "Hydroxy-beta-methylbutyrate" OR "Hydroxy-beta-methylbutyrate supplementation" OR "β-Hydroxy β-Methylbutyrate" OR "Beta-Hydroxy Beta-Methylbutyrate" OR "Ca-HMB" OR "FA-HMB" OR "Calcium HMB" OR "HMB free acid" OR "Beta-hidroxi-beta-metilbutirato") AND ("Resistance Training" OR "Strength Training" OR "Progressive Resistance Training" OR "Strength Exercise" OR "Resistance Exercise" OR "Resistance Strength Training" OR "Power Training" OR "Physical Exercise" OR "Weight Training" OR "Force Training" OR "Fitness Training" OR "Impact Exercise" OR "Elastic Resistance" OR "Elastic Exercise" OR "Loading" OR "Progressive" OR "High Speed")

ScienceDirect Search Formula: 399

("hmb" OR "Hydroxy-beta-methylbutyrate" OR "β-Hydroxy β-Methylbutyrate" OR "Beta-hidroxi-beta-metilbutirato") AND ("Resistance Training" OR "Strength Training" OR "Strength Exercise" OR "Resistance Exercise")
