# Supplementary material for: The Role of HMB Supplementation in Enhancing the Effects of Resistance Training in Older Adults: A Systematic Review and Meta-Analysis on Muscle Quality, Body Composition, and Physical Function
Source: Nutrients. 2025 Nov 20;17(22):3624. doi: 10.3390/nu17223624 (PMC12655442; doi:10.3390/nu17223624)

Supplementary Data 2. Funnel plot.

Funnel plot for muscle quality

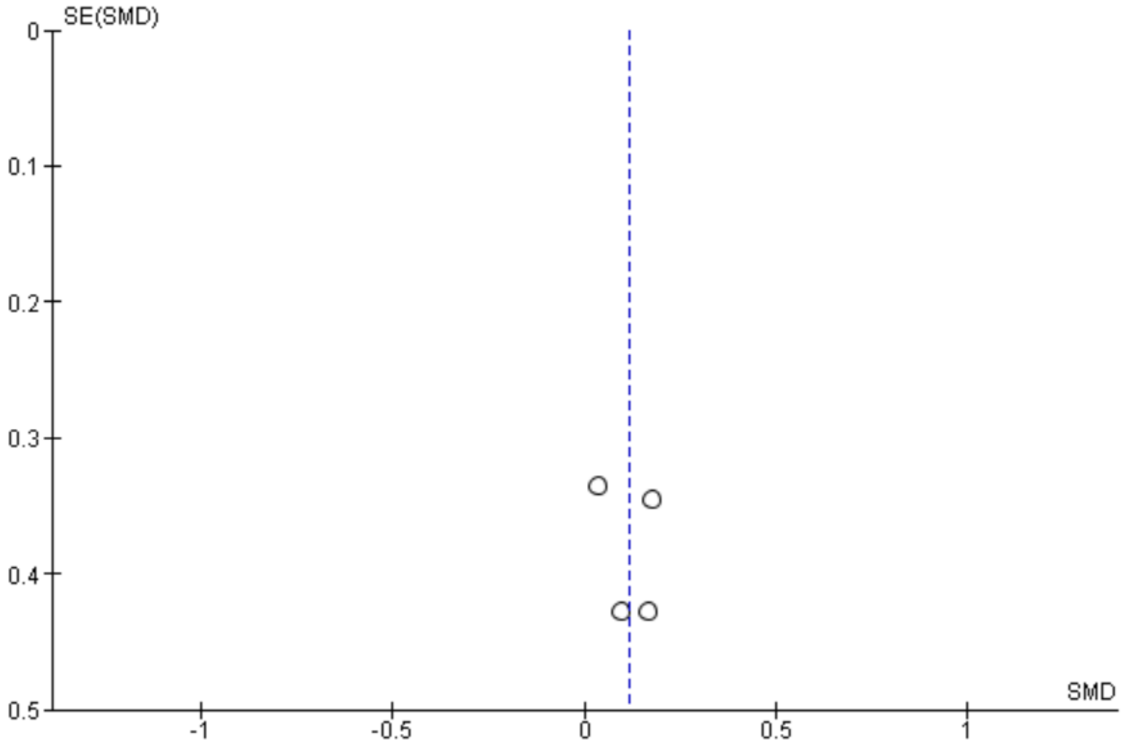

Funnel plot for handgrip strength

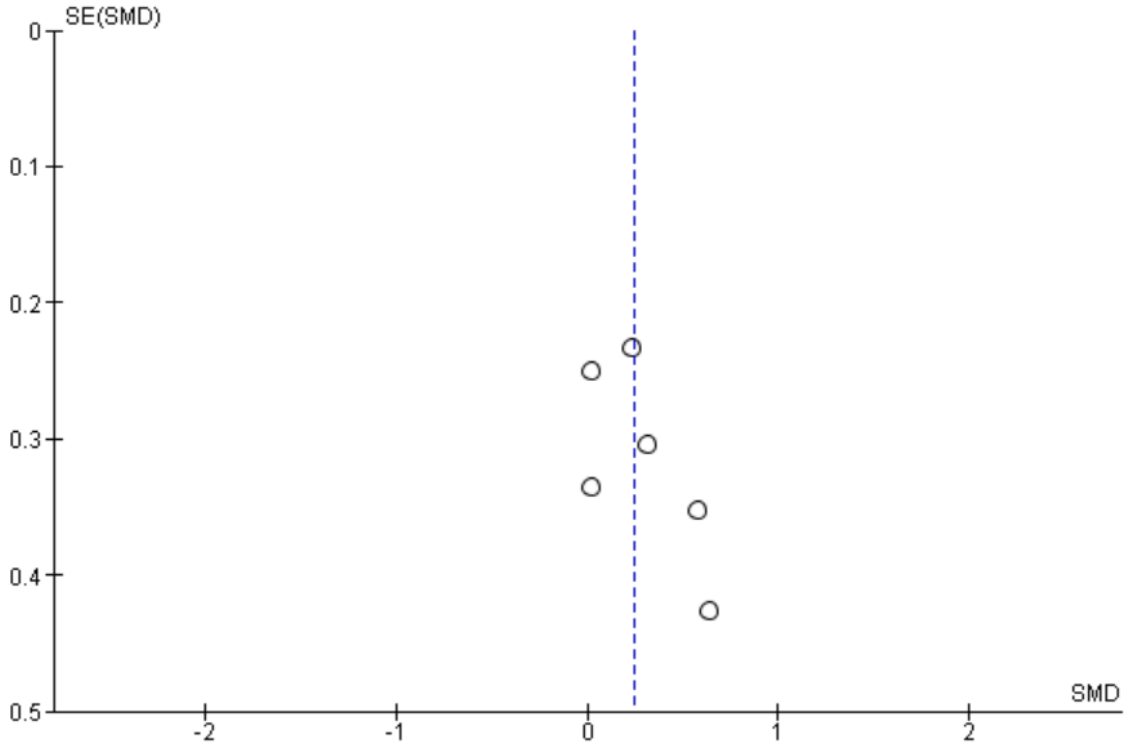

Funnel plot for gait speed

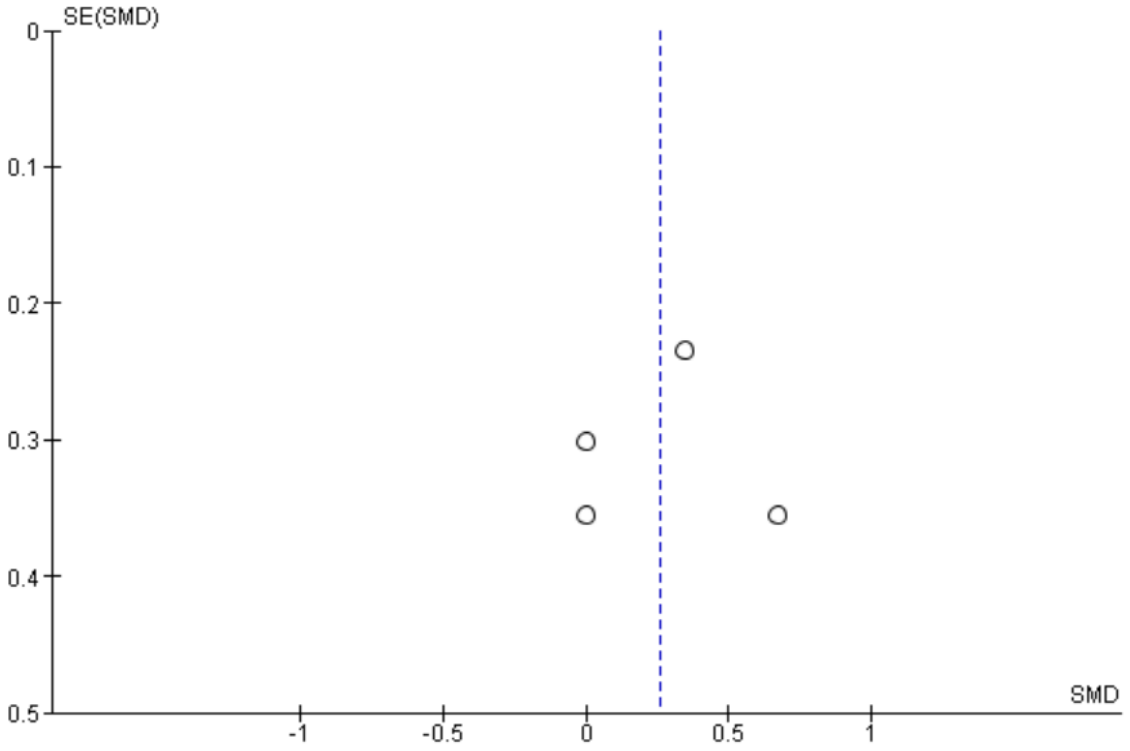

Funnel plot for SPPB

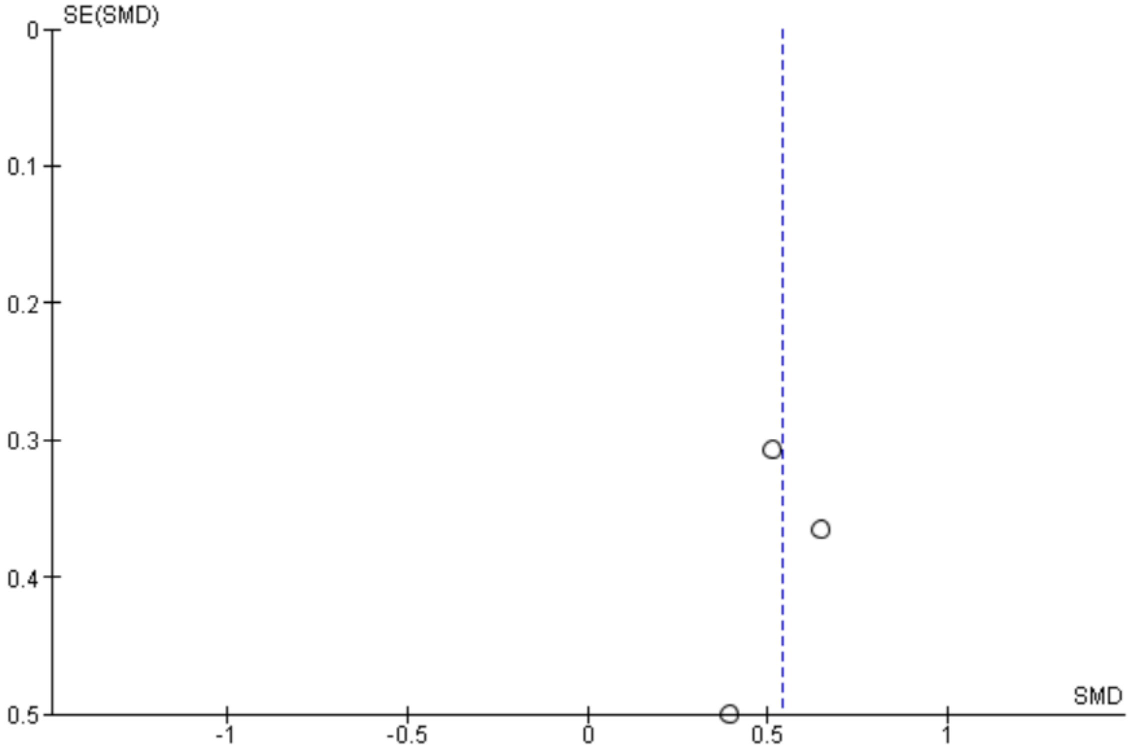

Funnel plot for appendicular lean mass

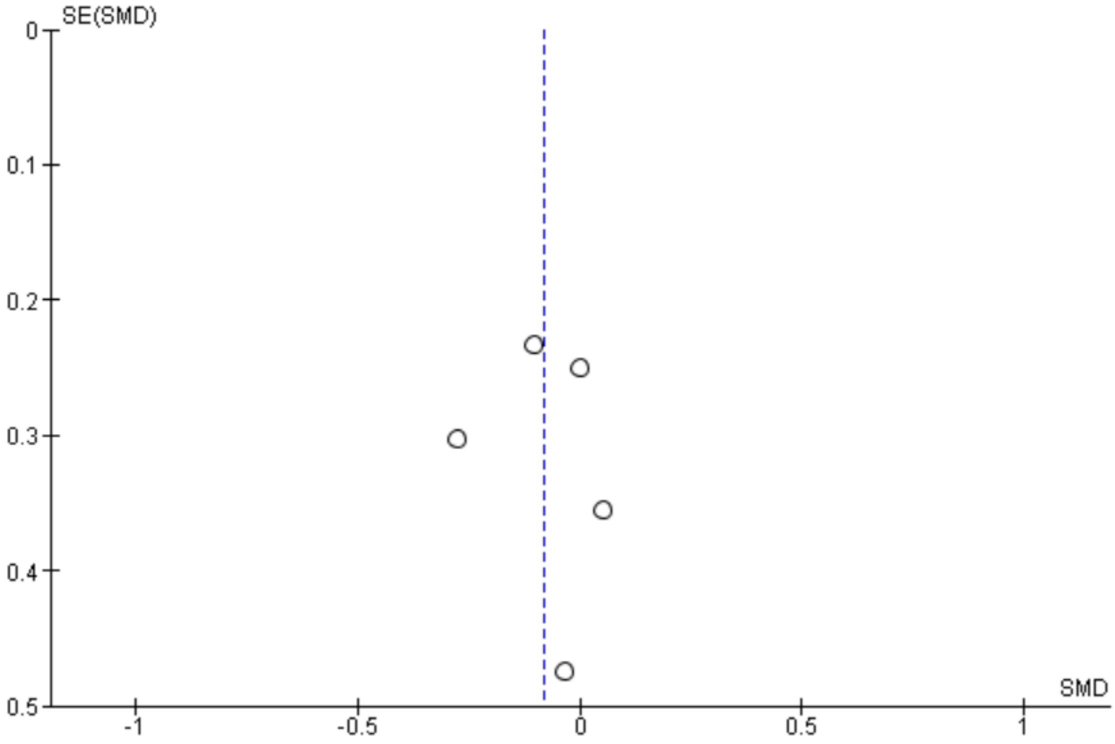

Funnel plot for fat mass

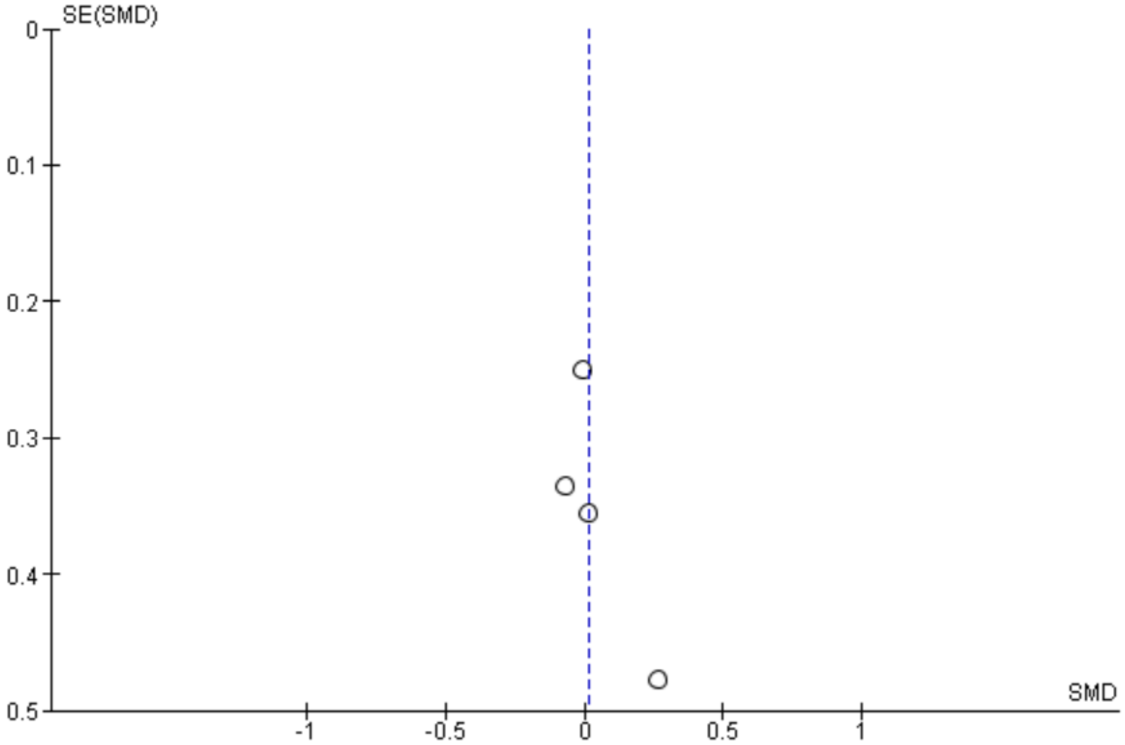

Funnel plot for body weight

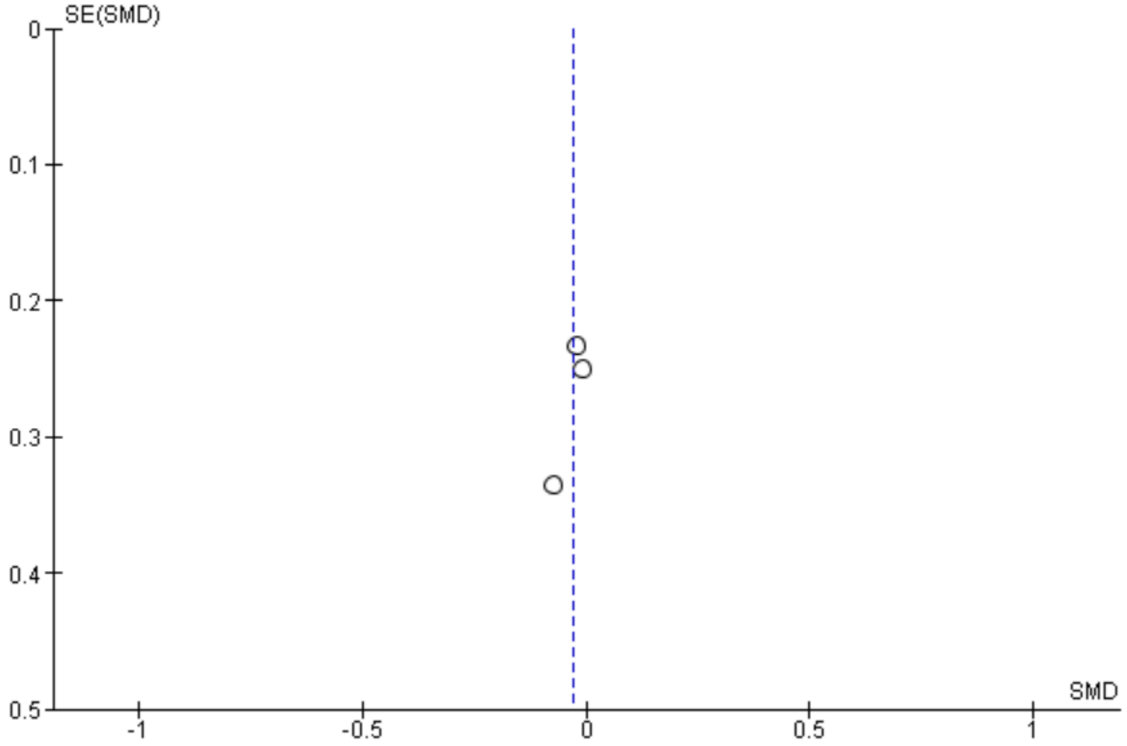

Supplement: Supplementary file 1 [file nutrients-17-03624-s001.zip › Supplementary Data 2.pdf]
